# Macrophage‐based delivery of anti‐fibrotic proteins alleviates bleomycin‐induced pulmonary fibrosis in mice

**DOI:** 10.1002/btm2.10555

**Published:** 2023-06-02

**Authors:** Huiying Liu, Cuiping Yang, Yun Gao, Xueli Zhang, Min Wang, Xinting Yu, Weidong Wang, Lixin Xie, Ping Tang, Xiushan Yin, Changqing Bai, Luo Zhang

**Affiliations:** ^1^ College of Pulmonary and Critical Medicine, The 8th Medical Centre Chinese PLA General Hospital Beijing China; ^2^ Medical School of Chinese PLA Beijing China; ^3^ College of Pharmaceutical and Biological Engineering Shenyang University of Chemical Technology Shenyang Liaoning China; ^4^ Department of Pathology The 5th Medical Centre, Chinese PLA General Hospital Beijing China; ^5^ Graduate School of Dalian Medical University Dalian Liaoning China; ^6^ Department of Respiratory and Critical Care Medicine 307 Clinical College, Anhui Medical University Beijing China; ^7^ Research Center of Bioengineering The Medical Innovation Research Division of Chinese PLA General Hospital Beijing China; ^8^ Department of Respiratory Shenzhen University General Hospital, Shenzhen University Clinical Medical Academy Shenzhen China; ^9^ RocRock Biotechnology (Shenzhen) Co., Ltd. Shenzhen China

**Keywords:** bleomycin, CD147, engineered macrophages, idiopathic pulmonary fibrosis, IL‐10, TGF‐β

## Abstract

Idiopathic pulmonary fibrosis (IPF) is a fatal interstitial lung disease characterized by chronic, progressive, and fibrotic lung injury. Although remarkable progress has been made toward understanding the pathogenesis of PF, finding more effective treatments for this fatal disease remains a challenge. In this study, we describe an innovative macrophage‐based approach to deliver anti‐fibrotic protein to the lung and inhibit PF in a mouse model of bleomycin (BLM)‐induced lung injury. We engineered macrophages to continuously secrete three types of proteins: interleukin‐10, which prevents inflammation; TGFRcFc, a soluble truncated TGF‐βR2 that blocks TGF‐β; and CD147, which induces matrix metalloproteinases (MMPs) and causes collagen degradation. Infusing these engineered macrophages into the lungs of BLM‐induced PF mouse models in an optimal pattern significantly ameliorated PF in mice. Specifically, the most effective therapeutic outcome was achieved by infusing IL‐10‐secreting macrophages on day 1, followed by TGFRcFc‐secreting macrophages on day 7 and CD147‐secreting macrophages on day 14 into the same mice after BLM treatment. Our data suggest that macrophage‐based delivery of anti‐fibrotic proteins to the lungs is a promising therapy for fibrotic lung disorders.

## 1 INTRODUCTION

Idiopathic pulmonary fibrosis (IPF) is a devastating interstitial lung disease characterized by the recruitment of inflammatory cells, deposition of excessive extracellular matrix (ECM), and destruction of the lung parenchyma through poorly understood mechanisms.^1^ Despite extensive research efforts in experimental and clinical studies, IPF remains an increasing cause of morbidity and mortality with an average survival rate of fewer than 3 years from diagnosis.^2^ Therefore, investigating the pathogenesis of IPF and developing new therapeutic methods for IPF patients are urgent needs.

According to the current paradigm, the main pathological features of patients with IPF and murine models of PF induced by bleomycin (BLM) include epithelial injury,^3^ initiation of an inflammatory response,^4^ transformation of fibroblasts into myofibroblasts, and finally excessive production of fibrillary ECM proteins, such as collagen and fibronectin.^5^ Lung injury in murine models of BLM‐induced PF is similar to human IPF. BLM causes inflammation within a short period of time, followed by a stage of increased expression of pro‐fibrotic factors and later, ECM accumulation.^6^ Treatment during the first 7 days would be considered “preventive” while treatment during the later stages after days 7–10 would be considered “therapeutic.”^7^ Therefore, implementing diverse treatment strategies for each stage of BLM‐induced fibrosis may lead to better outcomes.

Interleukin‐10 (IL‐10) is a cytokine with potent anti‐inflammatory and anti‐fibrotic properties, making it an appealing therapeutic candidate for IPF.^8^, ^9^ However, its therapeutic application is limited by its very short half‐life (1–2 min) in vivo.^10^ To achieve optimal results a steady and proper release of IL‐10 directly into lung tissue is necessary. Resident lung fibroblasts‐derived myofibroblasts are the major contributors to the processes of ECM deposition and tissue distortion in IPF.^11^ Under the effect of stimulation with fibrotic factors, mainly transforming growth factor‐β1 (TGF‐β1), resident fibroblasts in the lung lesion transform into myofibroblasts. TGF‐β1 binds to the extracellular domain of the TGF‐β type II receptor (Ex‐TβRII) and activates the downstream signal transduction. Blocking the binding of TGF‐β1 to Ex‐TβRII, by means of administration of soluble TGF‐β type II receptor (sTβR)^12^ or the anti‐TGF‐β1 antibody,^13^ has become an option for ameliorating PF. However, achieving adequate delivery to the damaged lungs without unacceptable systemic effects remains challenging. Therefore, a more specific approach is necessary to deliver sTβR effectively.

The synthesis and degradation of ECM are mainly regulated by matrix metalloproteinase (MMPs) and tissue inhibitors of metalloproteinases (TIMPs). Macrophages are an important source of MMPs.^14^ Kupffer cells (KCs) can express a variety of MMPs, such as MMP‐9, MMP‐12, and MMP‐13, to degrade the matrix, which is beneficial in the repair of liver damage and fibrosis.^15^, ^16^ Some studies have shown that the infusion of bone marrow‐derived macrophages in mice can significantly alleviate liver fibrosis and improve liver function.^17^ CD147, a membrane molecule that is essential for ECM remodeling via induction of MMPs,^18^ can be activated in macrophages to degrade ECM.^19^

Some studies have shown that macrophage‐transplantation therapy is a promising strategy for the treatment of lung diseases in which macrophages play a critical role. For instance, exogenous macrophage transplantation has been shown to be effective in murine models of alveolar proteinosis.^20^, ^21^ Transplanted macrophages engraft and persist long‐term in the alveolar space, and acquire an alveolar macrophage (AM) phenotype.^21^ Moreover, in our previous work, the transplantation of IL‐4‐secreting macrophages was also demonstrated to alleviate Lipopolysaccharides (LPS)‐induced lung inflammation and injury in mice.^22^

In the present study, we engineered macrophages to continuously secrete IL‐10, sTGFR‐Fc, or CD147. Infusing these cells into the lung in an optimal pattern improved the therapeutic effects on mice with BLM‐induced PF. Our data suggest that macrophage‐based delivery of anti‐fibrotic proteins to the lung is a promising therapeutic approach for fibrotic lung disorders. This study offers a practical strategy for macrophage‐based cargo delivery in the treatment of pulmonary fibrosis.

## 2 RESULTS

### 2.1 Establishment and characteristics of engineered macrophages

To establish engineered macrophages that secrete or express anti‐fibrotic factors, the RAW264.7 murine macrophage cell line was selected, because it has been widely used as a carrier in macrophage‐based therapy in mice.^23^, ^24^, ^25^ To achieve this, we set up engineered macrophages that constitutively secrete anti‐fibrotic proteins through lentivirus infection. First, a murine Myc‐targeted IL‐10‐secreted RAW264.7 cell line, referred to as IL10‐M, was constructed. Next, to target TGF‐β directly and inhibit BLM‐induced PF, a Myc‐tagged fusion protein consisting of an extracellular truncated domain of TGBβ‐Receptor 2 fused to IgG Fc domain was constructed, which was stably expressed in RAW264.7 cells and named TGFRcFc‐M. Given that CD147 plays a role in collagen deposition progression in PF, we engineered macrophages overexpressing Myc‐CD147, which was named CD147‐M. To use as a control, we also established a parallel green fluorescent protein (GFP)^+^ RAW264.7 cell line (Con‐GFP) that did not express therapeutic factors using the same backbone of the lentivirus vector (Figure S1a). The expression of Myc‐IL‐10, Myc‐TGFRcFc, and Myc‐CD147 in IL10‐M, TGFRcFc‐M, and CD147‐M was determined by flow cytometry (FCM), respectively. The FCM results showed that almost all of the cells were Myc positive, indicating the high purity of engineered cells. Then, we carried out qPCR assay to determine whether these factors influence macrophage polarization. The results in Figure S1c indicated that overexpression of IL‐10 and TGFRcFc elevated the expression of M1 marker CD80 and NOS2 but had no significant effect on M2 marker expression.

### 2.2 Infusion of unmodified RAW264.7 macrophages in the early phase accelerates BLM‐induced PF

First, we investigated the effect of unmodified macrophage infusion on BLM‐induced PF. To improve the stability of this nasal‐inhalation model, BLM was administered twice on two successive days with the last day being marked as day 0. As shown in the Figure S2a, b, there was an increase in microcomputed tomography (CT), Ashcroft score, and mRNA levels of fibrotic markers *COL1A1* and *FSP‐1* in mice after BLM challenge over time, indicating the success of BLM‐induced pulmonary fibrosis.

**FIGURE 1:**
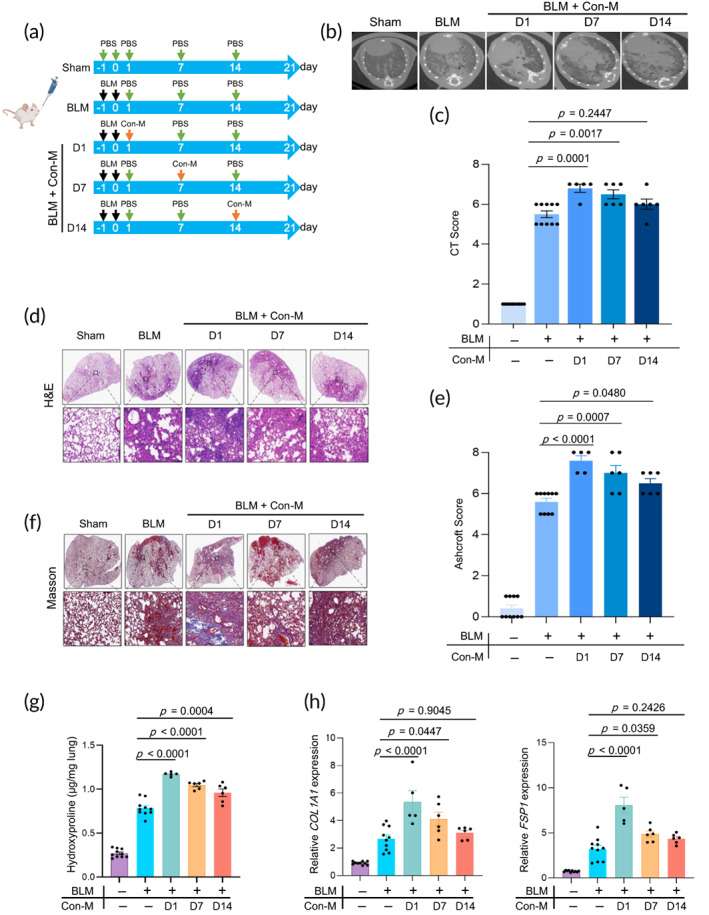
Unmodified RAW264.7 macrophage infusion at the early phase accelerates BLM‐induced PF. (a) Experimental design timeline of Con‐M administration on BLM‐induced lung injury (IPF). (b) Microcomputed tomography (micro‐CT) images and (c) CT fibrosis scores are presented. (d) Histological assessment of Con‐M treatment on IPF, representative photomicrographs of hematoxylin and eosin (H&E) staining, and (e) Ashcroft score of lung sections from sham control and BLM‐challenged mice with the indicated treatment. The inset shows a 10× image of the lung lobe. (f) The evaluation of collagen deposition by Masson's trichrome staining of lung tissue sections. The inset shows a 10× image of the lung lobe. (g) The content of hydroxyproline expressed in lung tissues (*n* = 5–10). (h) The mRNA levels of *COL1A1* and *FSP‐1* in the lungs were analyzed by RT‐PCR (*n* = 5–10).). All experiments were performed at least three times. Bars indicate the means ± standard error of the means.

To investigate the effects of cell infusion at different stages of BLM‐induced fibrosis, five groups of mice were established. The mice in the BLM group received PBS intranasal infusions on days 1, 7, and 14 after BLM administration. The mice in the BLM‐Con‐M 1 day group received intranasal infusions of Con‐M on day 1 and PBS on days 7 and 14. The BLM‐Con‐M 7 day group was infused with Con‐M on day 7 and PBS on days 1 and 14, while those in BLM‐Con‐M 14 days were infused with Con‐M on day 14 and PBS on days 1 and 7. The control group consisted of mice in the Sham group that were administrated on PBS only on days −1, 0, 1, 7, and 14 without BLM administration (Figure 1a). The body weights of all mice were measured every 7 days. On day 20, all the mice were scanned using a microcomputed tomography (CT) device. Unlike those of the PBS group, the micro‐CT images of mice in the BLM group showed typical PF imaging features, suggesting the establishment of BLM‐induced PF in mice. On day 21, all mouse groups were sacrificed and their lungs were isolated for subsequent assays.

For the purpose of detecting engineered cells in mice tissue, we have established a qPCR‐based engineered cell detecting method by amplifying BSD (blasticidin S deaminase) region of the inserted lentiviral vector that has been integrated into the genome of the engineered cells. The specificity of the established method was evaluated in cultured RAW264.7 infected and control cells. The qPCR data showed a strong amplification signal in the lung infused with engineered cells after 5 days, but no significant difference was found in the heart and liver (Figure S2d). This suggests that the engineered cells were undetectable in the heart and liver but resident in the lung.

Then, we observed that the mice in the Con‐M 1 day and 7 day groups showed a significant increase in body weight loss and lung weight compared to mice in the BLM group (Figure S2e, f). The micro‐CT images and corresponding scores of the lungs of mice infused with Con‐M on days 1 and 7 showed more severe PF compared to those in the PBS‐BLM group (Figure 1b, c). Hematoxylin and eosin (H&E) staining and corresponding Ashcroft score (Figure 1d, e) as well as Masson's trichrome staining (Figure 1f) of the lung sections from mice in the Con‐M 1 day and 7 day groups also indicated increasing fibrotic lesions. Notably, mice in the BLM‐Con‐M 1 day and 7 day groups presented excess collagen deposition, as evidenced by increased levels of hydroxyproline (HYP) (Figure 1g). To further quantitatively determine whether the infusion of Con‐M could promote the expression of fibrotic markers, we evaluated the mRNA expression levels of fibrotic genes by real‐time polymerase chain reaction (RT‐PCR) in the lungs of BLM‐treated mice. As illustrated, the expression of the fibrotic marker *COL1A1* and *FSP1* mRNA was higher in mice in the BLM‐Con‐M 1 day and 7 day groups but not in those of the 14 day group (Figure 1h). These results suggest that the infusion of unmodified RAW264.7 cells accelerates BLM‐induced PF, especially in the early phase. This observation can be partly attributable to the role of initiators and amplifiers of unmodified macrophages at the early inflammatory stage of BLM‐induced fibrosis. Therefore, anti‐inflammatory‐engineered macrophages should be used at this stage.

### 2.3 Infusion of IL‐10‐macrophages in the early phase suppresses BLM‐induced PF

**FIGURE 2:**
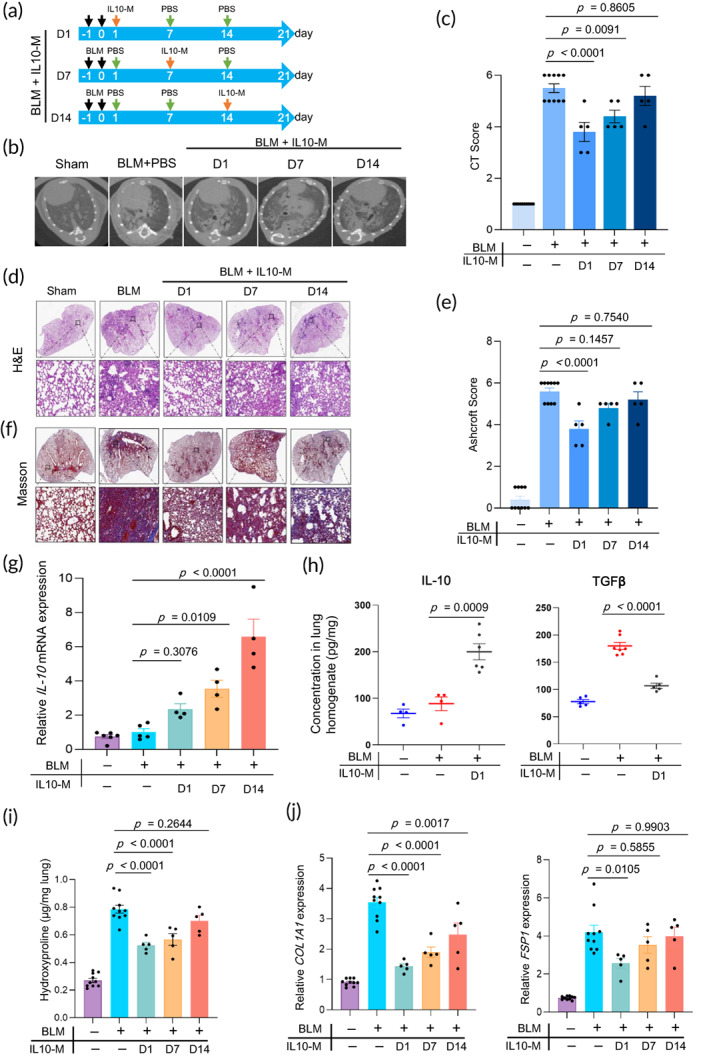
IL‐10‐macrophages infusion at the early phase suppresses BLM‐induced PF. (a) Experimental design timeline of IL10‐M administration on BLM‐induced lung injury (IPF). (b) Microcomputed tomography (micro‐CT) images and (c) CT fibrosis scores are presented. (d) Histological assessment of IL10‐M treatment on IPF, representative photomicrographs of hematoxylin and eosin (H&E) staining and (e) Ashcroft score of lung sections from sham control and BLM‐challenged mice with the indicated treatment. The inset shows a 10× image of the lung lobe. (f) The evaluation of collagen deposition by Masson's trichrome staining of lung tissue sections. (g) The mRNA levels of IL‐10 in the lungs were analyzed by RT‐PCR (*n* = 5–7). (h) The concentrations of IL‐10 and TGF‐β in lung homogenates were determined on day 7 by ELISA. (i) The content of hydroxyproline expressed in lung tissues (*n* = 5–10). (G) The mRNA levels of *COL1A1* and *FSP‐1* in the lungs were analyzed by RT‐PCR (qPCR) (*n* = 5–10). All experiments were performed at least three times. Bars indicate the means ± standard error of the means.

Before infusion, the ability of IL10‐M to secrete IL‐10 was tested. IL‐10 levels in the IL10‐M supernatant increased over time in cell culture, reaching 2000 pg/mL after 12 h. In contrast, IL‐10 levels in the Con‐M supernatant were almost undetectable at all three‐time points (Figure S3a). Then, the IL10‐M cells were infused via the intranasal route as Figure 2a indicated and the therapeutic effect was evaluated. First, we found that IL10‐M infusion ameliorated the body weight loss in mice administered with BLM, particularly in those infused on day 1 (Figure S3b). Additionally, the weight of the right lungs of the IL10‐M mice groups was significantly lower than that of the PBS‐BLM mice group (Figure S3c). Compared with mice from the PBS‐BLM group, infusion of IL10‐M on days 1 and 7 reduced lung injury and pulmonary fibrosis more remarkably, as shown by the micro‐CT images and corresponding scores of the lungs (Figure 2b, c), H&E (Figure 2d, e) and Masson's trichrome staining (Figure 2f). The lungs of mice were then isolated and their biomolecular level was tested. As expected, the IL‐10 mRNA levels (Figure 2g) in the lung of mice from IL10‐M groups were significantly higher than those of the Sham and BLM‐PBS groups. An enzyme‐linked immunosorbent assay **(**ELISA) assay also indicated that the level of IL‐10 protein was more elevated in the lungs of mice in group 1 day of IL10‐M compared with those of the mice in the Sham and BLM‐PBS groups. Conversely, IL10‐M infusion decreased the TGF‐β levels in the lungs of the mice in group 1 day (Figure 2h). In addition, the severity of PF in groups 1 day and 7 day was lower than that in the Sham and BLM‐PBS groups, as shown by the lower HYP content (Figure 2i). Meanwhile, RT‐PCR analysis of lung homogenates revealed a significant reduction of *Col1A1* and *FSP1* expression in mice in the IL‐10‐M 1 day group (Figure 2j). However, unlike in the 1 day and 7 day groups, infusion of IL10‐M on day 14 had no significant therapeutic effect. We also find no significant change in pro‐fibrotic factor PDGF and anti‐fibrotic factor BMP‐7 protein level in the lung of mice infused IL10‐M (Figure S3d). These results indicate that the infusion of IL‐10‐secreting macrophages in the early phase, rather than later, can ameliorate BLM‐induced PF.

### 2.4 Infusing TGFRcFc‐expressing macrophages in the middle phase suppresses BLM‐induced PF

**FIGURE 3:**
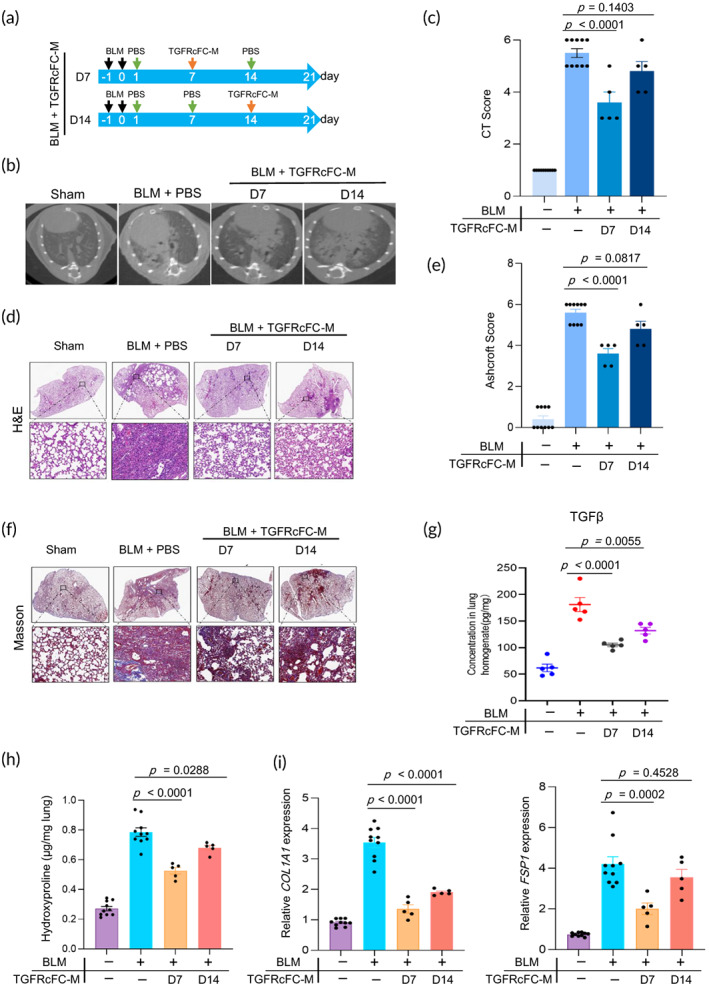
Protective effects of TGFRcFC‐M on BLM‐induced mouse model of IPF. (a) Experimental design timeline of TGFRcFC‐M administration on BLM‐induced lung injury (IPF). (b) Microcomputed tomography (micro‐CT) images and (c) CT fibrosis scores are presented. (d) Histological assessment of TGFRcFC ‐M treatment on IPF, representative photomicrographs of hematoxylin and eosin (H&E) staining and (e) Ashcroft score of lung sections from sham control and BLM‐challenged mice with the indicated treatment. The inset shows a 10× image of the lung lobe. (f) The evaluation of collagen deposition by Masson's trichrome staining of lung tissue sections. (g) The concentrations of TGF‐β were determined in lung homogenates by ELISA. (h) The content of hydroxyproline expressed in lung tissues (*n* = 5–10). (i) The mRNA levels of *COL1A1* and *FSP‐1* in the lungs were analyzed by RT‐PCR (qPCR) (*n* = 5–10). All experiments were performed at least three times. Bars indicate the means ± standard error of the means.

Before infusion, the inhibitory effect on TGF‐β of TGFRcFc‐M was tested. Western Blotting results revealed that Myc‐TGFRcFc was expressed (Figure S4a), which potently suppressed TGF‐β induced Smad2/3 phosphorylation in vitro (Figure S4b). As TGF‐β expression began at the intermediate stage of BLM‐induced PF, we canceled the infusion of TGFRcFc‐M on day 1 and evaluated the therapeutic effect as indicated in Figure 3a.

Although the infusion of TGFRcFc‐M did not ameliorate the body weight loss of BLM‐administered mice (Figure S4c), the weight of the right lungs of mice in the TGFRcFc‐M groups was remarkably lower than that of mice in the PBS‐BLM group (Figure S4d). As expected, attenuated lung injury and pulmonary fibrosis were observed in the TGFRcFc‐M 7 day group of mice, as shown by the micro‐CT images (Figure 3b, c), H&E (Figure 3d, e), and Masson's trichrome staining (Figure 3f). However, a slight but insignificant attenuated lung injury and pulmonary fibrosis were noted in the TGFRcFc‐M 14 day group of mice. Infusion of TGFRcFc‐M substantially decreased TGF‐β protein levels in the lungs of the 7 day mice group compared to those of the PBS‐BLM group (Figure 3g). In particular, the severity of PF was substantially lower, as shown by the lower HYP content (Figure 3h). Additionally, analysis of lung homogenates from mice in the TGFRcFc‐M 7 day group revealed a significant reduction in mRNA expression of both *Col1A1* and *FSP1*. Although, *Col1A1* mRNA expression was reduced in the lung of mice in the TGFRcFc‐M 14 day group, a slight but insignificant reduction in *FSP1* mRNA expression was observed in the same mice (Figure 3i). We also find a decreased PDGF, but not BMP‐7, protein level in the lung of mice infused with TGFRcFc‐M (Figure S3d). Collectively, our data suggest that infusion of TGFRcFc‐M at the intermediate stage can relieve BLM‐induced lung injury and fibrosis.

### 2.5 Infusing CD147‐expressing macrophages in the later phase ameliorates BLM‐induced PF

**FIGURE 4:**
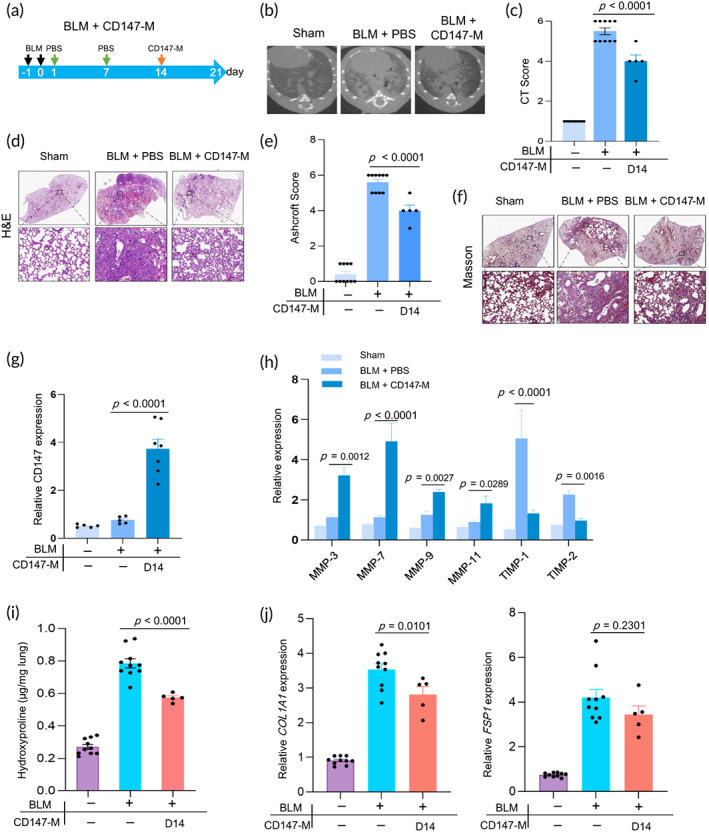
Protective effects of CD147‐M on BLM‐induced mouse model of IPF. (a) Experimental design timeline of CD147‐M administration on BLM‐induced lung injury (IPF). (b) Microcomputed tomography (micro‐CT) images and (c) CT fibrosis scores are presented. (d) Histological assessment of CD147‐M treatment on IPF, representative photomicrographs of hematoxylin and eosin (H&E) staining and (e) Ashcroft score of lung sections from sham control and BLM‐challenged mice with the indicated treatment. The inset shows a 10× image of the lung lobe. (f) The evaluation of collagen deposition by Masson's trichrome staining of lung tissue sections. (g) The mRNA levels of CD147 in the lungs were analyzed by RT‐PCR (*n* = 5–7). (h) The mRNA levels of MMPs in the lungs were analyzed by RT‐PCR (*n* = 5–7). (i) The content of hydroxyproline expressed in lung tissues (*n* = 8–10). (j) The mRNA levels of *COL1A1* and *FSP‐1* in lungs were analyzed by qPCR (*n* = 5–10). All experiments were performed at least three times. Bars indicate the means ± standard error of the means.

Before infusion, we measured the expression levels of MMPs that were induced by CD147. RT‐PCR results revealed that CD147‐M expressed higher levels of *CD147* (Figure S 5a) and *MMP*s, including *MMP‐3*, *MMP‐7*, and *MMP‐11* (Figure S5b). As collagen deposition occurs at a later stage, CD147‐M was infused into the lungs of mice with BLM‐induced fibrosis on day 14 alone, and the therapeutic effect was evaluated (Figure 4a).

Although the infusion of CD147‐M did not relieve the body weight loss of BLM‐administered mice (Figure S5c), the weight of the right lungs in the CD147‐M group was lower than that in the PBS‐BLM group (Figure S5d). Attenuated lung injury and PF were observed in the CD147‐M group as shown by the micro‐CT images (Figure 4b, c), H&E (Figure 4d, e), and Masson's trichrome staining (Figure 4f). The mRNA levels of *CD147*, as well as *MMP‐3*, *MMP‐7*, *MMP‐9*, and *MMP‐11*, were elevated in lung tissue from the CD147‐M group than that in the PBS‐BLM group (Figure 4g, h). Conversely, the expression of MMPs inhibitors *TIMP‐1* and *TIMP‐2* was decreased (Figure 4h). Additionally, the severity of PF in the CD147‐M group was reduced, as evidenced by the lower HYP content (Figure 4i). RT‐PCR results displayed a slight difference in the mRNA levels of *Col1A1* and no difference in *FSP1* between the CD147 and PBS groups (Figure 4j). We also observed a decrease in PDGF, but not BMP‐7, protein levels in the lung of mice infused with CD147‐M (Figure S3d). Collectively, these results suggest that the infusion of CD147‐M reduces BLM‐induced PF by accelerating collagen degradation.

### 2.6 Infusing combinatorial engineered macrophages has an elevated therapeutic effect on BLM‐induced PF

**FIGURE 5:**
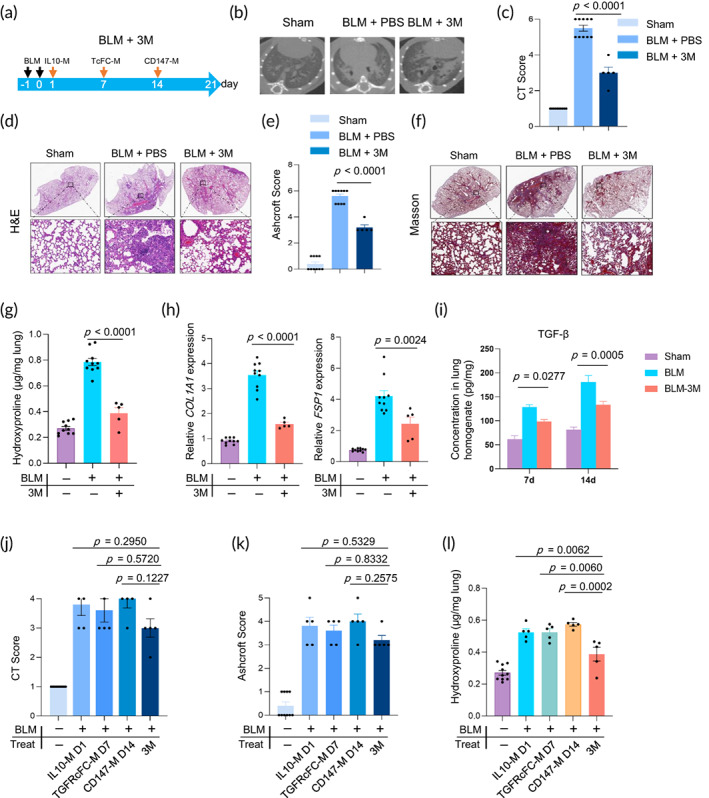
Protective effects of the combination of the 3 M infusion on BLM‐induced mouse model of IPF. (a) Experimental design timeline of 3 M administration on BLM‐induced lung injury (IPF). (b) Microcomputed tomography (micro‐CT) images and (c) CT fibrosis scores are presented. (d) Histological assessment of 3 M treatment on IPF, representative photomicrographs of hematoxylin and eosin (H&E) staining and (e) Ashcroft score of lung sections from sham control and BLM‐challenged mice with the indicated treatment. The inset shows a 10× image of the lung lobe. (f) The evaluation of collagen deposition by Masson's trichrome staining of lung tissue sections. (g) The content of hydroxyproline (HYP) expressed in lung tissues (*n* = 8–10). (h) The mRNA levels of *COL1A1* and *FSP‐1* in the lungs were analyzed by RT‐PCR (*n* = 5–10). (i) The concentrations of TGF‐β in lung homogenates were determined on days 7 and 14 by ELISA. (j) CT fibrosis scores and (k) Ashcroft score of lung sections from healthy control and BLM‐challenged mice with IL‐10‐M, TGFRcFC‐M, CD147‐M, or 3 M treatment. (l) Comparison of HYP content in the IL‐10‐M, TGFRcFC‐M, CD147‐M, or 3 M groups. All experiments were performed at least three times. Bars indicate the means ± standard error of the means.

Based on the above results, we sought to evaluate whether a combinatorial infusion strategy of engineered macrophages would have a stronger effect on BLM‐induced PF. The strategy is demonstrated in Figure 5a and is referred to as 3 M. Briefly, IL‐10‐M was infused on day 1, TGFRcFc‐M on day 7, and CD147 M on day 14 into the same mice after BLM administration. The therapeutic effect was then evaluated.

This strategy differs from the above single‐type macrophage infusion. Treatment of 3 M significantly reduced the body weight loss of BLM‐administered mice (Figure S 6a). Meanwhile, the weight of the right lungs of 3 M groups of mice was significantly lower than that of the PBS‐BLM group (Figure S6b). The micro‐CT images (Figure 5b, c), H&E (Figure 5d, e), and Masson's trichrome staining (Figure 5f) showed significantly attenuated lung injury and PF in the 3 M mice groups. Additionally, the severity of PF was lower, as shown by the lower HYP content (Figure 5g). Analysis of lung homogenates revealed a substantial reduction of the mRNA expression of *Col1A1* and *FSP1* (Figure 5h) and the protein level of TGF‐β (Figure 5i). Although the 3 M infusion did not result in a statistically significant reduction in the CT Score (Figure 5j) and H&E Ashcroft score (Figure 5k) but the HYP content of the 3 M group was lower than that of single groups (Figure 5l). Collectively, our data support that the combinatorial infusion of 3 M has an elevated therapeutic effect on BLM‐induced lung fibrosis.

## 3 DISCUSSION

The difficulty of delivery and short half‐life of protein drugs limit their application in disease treatment. In this study, we demonstrate that infusion of engineered macrophages expressing anti‐fibrotic proteins is efficient to alleviate BLM‐induced pulmonary fibrosis in mice. We first examined the effect of infusion unmodified macrophages, which do not express anti‐fibrotic protein, on pulmonary fibrosis in mice. The results showed that infusion of these macrophages during the early and middle stages of BLM‐induced fibrosis, especially on day 1, can aggravate pulmonary fibrosis. However, there was no significant effect on the formation of fibrosis when infusion was performed on day 14. These unexpected results may be due to the additional inflammatory factors and other pro‐fibrosis factors secreted by unmodified macrophages. Therefore, infusing anti‐inflammatory cells may be appropriate rather than other types of engineered macrophages at the early stage. Due to the potent anti‐inflammatory properties of IL‐10, we established IL10‐M and tested the effect of IL10‐M infusion on fibrosis. The results suggested that IL10‐M infusion on day 1 had the best inhibitory effect on pulmonary fibrosis among other groups. Since TGFRcFc‐M inhibits TGF‐β signaling rather than early inflammation, we only infused TGFRcFc‐M at the middle and late stages. We found that TGFRcFc‐M infusion at the middle stages manifested effective inhibition on fibrosis but had little inhibitory effect when infusion was performed at a later stage. Moreover, we observed upregulation of the expression of M1 markers CD80 and NOS2, in IL10‐M and TGFRcFc‐M. However, this upregulation did not affect their protein delivery function, possibly because the expression level was below the threshold for classical activation of M1 macrophages. Similarly, because the function of CD147 is to induce the degradation of collagen, we did not infuse CD147‐M at the early and middle stages, but only at the late stages and verified its inhibitory effect on fibrosis. Based on the above results of single engineered macrophage infusion, we designed a combinational therapy strategy known as 3 M. 3 M strategy was carried out with early infusion of IL10‐M, mid‐infusion of TGFRcFc‐M, and late infusion of CD147‐M. The 3 M strategy significantly reduced the degree of pulmonary fibrosis in mice compared with the control group and was superior to any single‐engineered macrophage therapy on the effect of HYP assay. Collectively, we propose a combination therapy with diverse engineered macrophages depending on the progression of pulmonary fibrosis to achieve better therapeutic outcomes.

In recent years, mesenchymal stem cells (MSCs)‐based therapy has received increasing attention in treating pulmonary fibrosis due to their multi‐lineage differentiation potential, migratory ability, and self‐renewal properties. Several studies have shown that MSCs can suppress inflammation, relieve fibrosis, and prolong the survival time in preclinical models of PF induced by BLM, silica, paraquat, or radiation.^26^, ^27^, ^28^, ^29^, ^30^, ^31^, ^32^ However, macrophage‐based therapy has its own advantages. First, macrophages, such as AMs, are resident cell types in the lung, which suggests a compatible tissue microenvironment for the survival and maintenance of infused macrophages. Second, infused macrophages are able to alter their phenotype to adapt to the local tissue microenvironments and reshape their behavior, making them more compatible to work like the tissue‐resident macrophages in infiltrated tissue. Additionally, infusion of engineered macrophage had no observable effect on monocyte and granulocyte recruitment (Figure S7). These aforementioned characteristics, combined with the widely approved secretory ability, make macrophages a good protein delivery vector. Furthermore, the flexible transformation from pro‐inflammatory to anti‐inflammatory or tissue‐repaired phenotype of macrophages make them not only a vector but also a real effector in dealing with fibrosis. The infusion of engineered macrophages can play dual roles and achieve a better therapeutic effect.

Although this study demonstrates a proof of concept for genetically engineered macrophages for the treatment of PF. Additional work is necessary to translate and evaluate the efficacy of this approach in humans. In our studies, we used RAW264.7 cells, a leukemia‐derived population of cells that are easy to culture and operate, but their potential safety has not yet been elucidated. Therefore, primary macrophages should be used for clinical purposes. In addition, as mentioned above, infusion of unmodified macrophages may exacerbate IP. Therefore, high transfection efficiency is needed to avoid this outcome. Furthermore, persistent exposure to secreted protein would be harmful. For instance, CD147 is found to involve in the regulation of tumor microenvironment and cancer progression by controlling glycolysis beyond by its well‐known ability to induce proteinases that lead to matrix degradation, tumor cell invasion, metastasis, and angiogenesis.^33^ Therefore, it is necessary to eliminate the expression of CD147 immediately after collagen degradation is complete. Hence, an inducible expression system and/or a turnoff strategy should be developed and implemented in future investigations.

## 4 CONCLUSION

In this study, we engineered macrophages to continuously secrete interleukin‐10, TGFRcFc, and CD147, respectively. Nasal‐inhalation of these engineered macrophages into the lung of mice at certain stages after treatment of BLM significantly ameliorated PF. Furthermore, we established a combined optimized cell infusion strategy for different stages of fibrosis, which achieved an ideal therapeutic efficacy. Collectively, these results demonstrate that macrophage‐based anti‐fibrotic protein delivery to the lungs is an efficient method to ameliorate PF induced by BLM in mice and suggest a potential for translational applications in the future.

## 5 MATERIALS AND METHODS

### 5.1 Mice model

Male, healthy, BALB/c mice (6–8 weeks old, specific pathogen‐free) were purchased from Beijing Huafukang Bioscience Co. Inc. (Beijing, China). All experiments involving animal subjects were performed in accordance with guidelines approved by the Chinese Academy of Military Medicine Science Animal Care and Use Committee at Beijing (IACUC: DWZX‐2019‐012). Mice were anesthetized via controlled isoflurane flow. PF was established in mice models by intranasal instillation of BLM‐Sulfate (NSC125066, Selleck Chemicals, Houston, TX, USA) (5 mg/kg dissolved in PBS, 50 μL). Mice received nasal inhalations of 1∙10^5^ engineered macrophage cells per g mouse weight in 50 μL of sterile PBS, while control mice received 50 μL of sterile PBS on the day as indicated.

### 5.2 Cell culture

RAW264.7, MLE‐12, HEK293T cells were cultured in Dulbecco's modified Eagle's medium (DMEM, Gibco) supplemented with 10% (v/v) fetal bovine serum (FBS, Gibco), 100 μg/mL penicillin, and 100 μg/mL streptomycin.

### 5.3 Establishing engineered macrophages

The lentiviral constructs expressing murine IL‐10, CD147, and TGFRcFC were cloned into the pCDH vector (SBI). Lentiviral vectors were transfected with packing plasmids into 293 T cells, and the viral particles were collected and used to infect RAW264.7 cells. The selection was carried out by culturing cells in a medium containing 5 μg/mL puromycin for 2 days.

### 5.4 Western blot

Cells were harvested and extracted using RIPA buffer. Lung tissues from mice were homogenized in an appropriate amount of RIPA buffer. Total protein was quantified using a BCA (Bicinchoninic acid) protein assay reagent kit (Tiangen, China). Equal amounts of proteins were separated on a 10% SDS‐PAGE gel and transferred onto a PVDF membrane (PALL, USA). Membranes were incubated with anti‐Smad2/3, anti‐pSmad2/3 (Cell Signaling Technology, USA), anti‐Myc (Medical & Biological Laboratories Co, Japan), anti‐β‐actin (Santa Cruz Biotechnology, USA), and anti‐GAPDH (Santa Cruz Biotechnology, USA) overnight at 4°C. After three washes in 1 × TBST buffer, the blots were incubated with either horseradish peroxidase‐conjugated goat anti‐rabbit antibody or horseradish peroxidase‐conjugated goat anti‐mouse antibody (1:2000; Jackson Immuno Research, USA) for 1 h at room temperature, rewashed, and then developed using an ECL chemiluminescence kit (Thermo Fisher Scientific, USA).

### 5.5 Quantitative PCR analysis

Total RNA was isolated with TIANGEN (RNAprep pure Cell/Bacteria Kit), and reverse transcription was performed with ReverTra Ace (Toyobo) according to the manufacturer's instructions. cDNA fragments were synthesized by RT‐PCR Master Mix (Toyobo); fluorescence for each gene was detected on an Agilent Mx3005P qPCR System (Agilent, USA). Target gene expression levels were normalized to the housekeeping gene, β‐actin, and fold change was calculated using the 2^−ΔΔCT^ method. The primers used for RT‐PCR were as follows:

*COL1A1*: forward 5′‐ GGAGGGAACGGTCCACGAT −3′.

reverse 5′‐ GAGTCCGCGTATCCACAA ‐3′;

*FSP‐1*: forward 5′‐ AGGCAACGAGGGTGACAAGTTC ‐3′.

reverse 5′‐ CATCATGGCAATGCAGGACAG ‐3′;

*CD147*: forward 5′‐ GGAATGCTCCAAACGACAG −3′.

reverse 5′‐ CCCATCAACAGAGAGCGA ‐3′;

*MMP‐2*: forward 5′‐AGATTGATGCCGTGTACGAGG‐3′.

reverse 5′‐TCCAGGAGTCTGCGATGAGC ‐3′;

*MMP‐3*: forward 5′‐CAGGCATTGGCACAAAGGTG‐3′.

reverse 5′‐GTGGGTCACTTTCCCTGCAT‐3′;

*MMP‐7*: forward 5′‐ TGGTACCATAATGTCCTGAATG‐3′.

reverse 5′‐ TCGTTATTGGCAGGAAGCACACAATGAATT‐3′;

*MMP‐9*: forward 5′‐GCTGACTACGATAAGGACGGC‐3′.

reverse 5′‐ AGGAAGACGAAGGGGAAGACG‐3′;

*MMP‐11*: forward 5′‐ CCGGAGAGTCACCGTCATC‐3′.

reverse 5′‐ GCAGGACTAGGGACCCAATG‐3′;

*TIMP‐1*: forward 5′‐ GACCTGGTCATAAGGGCTAAA‐3′.

reverse 5′‐ GCCCGTGATGAGAAACTCTTGACT‐3′;

*TIMP‐2*: forward 5′‐ TCAGAGCCAAAGCAGTGAGC‐3′.

reverse 5′‐ GCCGTGTAGATAAACTCGATGTC‐3′;

*BSD*: forward 5′‐ TCGTCGCGATCGGAAATGAG‐3′.

reverse 5′‐ GATCGAGAAGCACCTGTCGG‐3′;

### 5.6 Measurement of HYP

The measurement of HYP was conducted with an HYP measurement kit (Nanjing Jiancheng Bioengineering Institute, Nanjing, China) according to the manufacturer's instructions.

### 5.7 ELISA

The levels of cytokines from the supernatant of whole lung homogenate were measured using commercially available mouse IL‐10, TGF‐β1 ELISA kits (R&D Systems Inc., Minneapolis, MN, USA), PDGF, BMP7 (Boster). The experiment was repeated three times, and the cytokine concentrations were calculated using standard curves.

### 5.8 Histopathological observation and lung injury scores

Morphological analysis of mouse lung was performed by micro‐CT scanning (Quantum FX Demo, PerkinElmer‐Caliper LS, MA, USA) and H&E staining. The entire left lower lobe of the lung was fixed in 4% formaldehyde neutral buffer solution for 48 h, dehydrated in a graded ethanol series, embedded in paraffin, and sliced into 5‐μm sections. These sections were then stained with H&E or with Masson's trichrome for histopathological analysis. Two pathologists blinded to the treatment regimen were invited to assess the severity of lung injury using the following histological features: edema, hyperemia and congestion, neutrophil margination and tissue infiltration, intra‐alveolar hemorrhage and debris, and cellular hyperplasia. A scoring system was used to grade the degree of lung injury. Each feature was graded as absent, mild, moderate, or severe, and was assigned a score from 0 to 8. The total score was calculated for each mouse.^34^

### 5.9 Computed tomography

We performed the radiological evaluation of the chest using a micro‐CT (Quantum FX Demo, PerkinElmer‐Caliper LS, MA, USA). Mice received isoflurane inhalation as anesthesia and were placed in a prone position for data acquisition.^35^ Three specialists in respiratory disease blinded to the treatment groups scored the chest CT findings based on the following criteria: score 1, normal lung findings; 2, intermediate findings; 3, slight lung fibrosis; 4, intermediate findings; 5, moderate lung fibrosis; 6, intermediate findings; and 7, advanced lung fibrosis.^35^

### 5.10 Flow cytometry analysis

Engineered RAW264.7 cells and alveolar lavage fluid cells were washed with PBS, then resuspended with 0.5% bovine serum albumin in PBS. Cells were incubated in antibody dilution to detect CD11b (BD, USA), Ly6C antibody (BD, USA), F4/80 (eBioscience, USA), Ly6G (eBioscience, USA), and Myc (Cell Signaling Technology, USA) according to the manufacturer's instructions. Data were acquired using flow cytometry (NovoCyte, ACEA Biosciences), and further analyses were performed in FlowJo (Version 7.6).

### 5.11 Statistical analysis

All analyses were performed using GraphPad (GraphPad Prism 9.0, San Diego, CA, US). Data were presented as the mean ± standard deviations (SD). Statistical significance was determined by one‐way analysis of variance and the student's *t*‐test for the comparisons between groups. A value of *p* < 0.05 was considered statistically significant.

## AUTHOR CONTRIBUTIONS

**Huiying Liu:** Conceptualization (equal); data curation (equal); funding acquisition (equal); resources (equal); writing – original draft (equal). **Cuiping Yang:** Data curation (equal); formal analysis (equal); validation (equal); writing – original draft (equal). **Yun Gao:** Data curation (equal); formal analysis (equal); methodology (equal); visualization (equal). **Xueli Zhang:** Methodology (equal); visualization (equal). **Min Wang:** Formal analysis (equal); methodology (equal). **Xinting Yu:** Data curation (equal); methodology (equal); validation (equal). **Ping Tang:** Methodology (equal). **Weidong Wang:** Resources (equal); writing – review and editing (equal). **Lixin Xie:** Resources (equal); supervision (equal). **Xiushan Yin:** Funding acquisition (equal); methodology (equal); project administration (equal); resources (equal); writing – review and editing (equal). **Changqing Bai:** Funding acquisition (equal); investigation (equal); project administration (equal); resources (equal); supervision (equal); writing – review and editing (equal). **Luo Zhang:** Conceptualization (equal); data curation (equal); funding acquisition (equal); investigation (equal); project administration (equal); resources (equal); supervision (equal); writing – original draft (equal).

## CONFLICT OF INTEREST STATEMENT

The authors declare that they have no conflict of interest.

## Supporting information


**Figure S1.** (a) Representative flow cytometry analysis of GFP+ cells. (b) Representative flow cytometry analysis of engineered cells by using a Myc‐FITC antibody. (c) The mRNA levels of indicted gens in engineered cells were analyzed by RT‐PCR (qPCR). All experiments were performed at least three times. Bars indicate the means ± standard error of the means.
**Figure S2.** (a) CT fibrosis scores are presented at the day after BLM treated. (b) Ashcroft score of lung sections are presented at the day after BLM treated. Body weight at day 21 after BLM treatment. (c) The mRNA levels of *COL1A1* and *FSP‐1* in lungs at the day after BLM treated were analyzed by RT‐PCR (qPCR) (d) The gDNA levels of blasticidin S deaminase (BSD) in lungs, liver, and heart from the mice infused Con‐M or not at the 5th day after BLM treated were analyzed by RT‐PCR (qPCR). (e) Changes in body weight between day 21 and day 0. (f) Right lung wet weight of mice from the indicated groups. All experiments were performed at least three times. Bars indicate the means ± standard error of the means.
**Figure S3.** (a) The level of IL‐10 in the supernatant of IL‐10‐M or Con‐M was determined by ELISA. (b) Changes in body weight between day 21 and day 0. (c) Right lung wet weight of mice from the indicated groups. (d) The concentrations of indicated cytokines were determined in lung homogenates after 5 days of BLM treatment by ELISA. All experiments were performed at least three times. Bars indicate the means ± standard error of the means.
**Figure S4.** (a) Western blot analysis of TGFRcFC protein in TGFRcFC and control RAW264.7 cells. (b) TGFRcFC‐M supernatant inhibits the TGF‐β/Smad signaling pathway. MLE and RAW264.7 cells were treated as indicated and the cells collected and Western blot analysis was used to detect p‐Smad2/3 and Smad2/3. (c) Changes in body weight between day 21 and day 0. (d) Right lung wet weight of mice from the indicated groups. All experiments were performed at least three times. Bars indicate the means ± standard error of the means.
**Figure S5.** (a) mRNA levels of CD147 or (b) MMPs in CD147‐M or Con‐M cells were analyzed by RT‐PCR. (c) Changes in body weight between day 21 and day 0. (d) Right lung wet weight of mice from indicated groups. All experiments were performed at least three times. Bars indicate the means ± standard error of the means.
**Figure S6.** (a) Changes in body weight between day 21 and day 0. (b) Wet weight of right lung from mice in the indicated groups (*n* = 8–10). The results are representative of at least three independent experiments. Bars indicate the means ± standard error of the means.
**Figure S7.** (a) Representative flow cytometry analysis of BAL cells from mice after 3 days the indicated cells were infused. (b) Total flow cytometry analysis of BAL cells from mice as (a). Bars indicate the means ± standard error of the means. The results are representative of at least three independent experiments.

## Data Availability

The data that support the findings of this study are available from the corresponding author upon reasonable request.
