# Supplementary material for: Macrophage‐based delivery of anti‐fibrotic proteins alleviates bleomycin‐induced pulmonary fibrosis in mice
Source: Bioeng Transl Med. 2023 Jun 2;8(5):e10555. doi: 10.1002/btm2.10555 (PMC10486326; doi:10.1002/btm2.10555)
Supplement: Supplementary file 1 — Figure S1. (a) Representative flow cytometry analysis of GFP+ cells. (b) Representative flow cytometry analysis of engineered cells by using a Myc‐FITC antibody. (c) The mRNA levels of indicted gens in engineered cells were analyzed by RT‐PCR (qPCR). All experiments were performed at least three times. Bars indicate the means ± standard error of the means. Figure S2. (a) CT fibrosis scores are presented at the day after BLM treated. (b) Ashcroft score of lung sections are presented at the day after BLM treated. Body weight at day 21 after BLM treatment. (c) The mRNA levels of COL1A1 and FSP‐1 in lungs at the day after BLM treated were analyzed by RT‐PCR (qPCR) (d) The gDNA levels of blasticidin S deaminase (BSD) in lungs, liver, and heart from the mice infused Con‐M or not at the 5th day after BLM treated were analyzed by RT‐PCR (qPCR). (e) Changes in body weight between day 21 and day 0. (f) Right lung wet weight of mice from the indicated groups. All experiments were performed at least three times. Bars indicate the means ± standard error of the means. Figure S3. (a) The level of IL‐10 in the supernatant of IL‐10‐M or Con‐M was determined by ELISA. (b) Changes in body weight between day 21 and day 0. (c) Right lung wet weight of mice from the indicated groups. (d) The concentrations of indicated cytokines were determined in lung homogenates after 5 days of BLM treatment by ELISA. All experiments were performed at least three times. Bars indicate the means ± standard error of the means. Figure S4. (a) Western blot analysis of TGFRcFC protein in TGFRcFC and control RAW264.7 cells. (b) TGFRcFC‐M supernatant inhibits the TGF‐β/Smad signaling pathway. MLE and RAW264.7 cells were treated as indicated and the cells collected and Western blot analysis was used to detect p‐Smad2/3 and Smad2/3. (c) Changes in body weight between day 21 and day 0. (d) Right lung wet weight of mice from the indicated groups. All experiments were performed at least three times. Bars indicate t [file BTM2-8-e10555-s001.pdf]

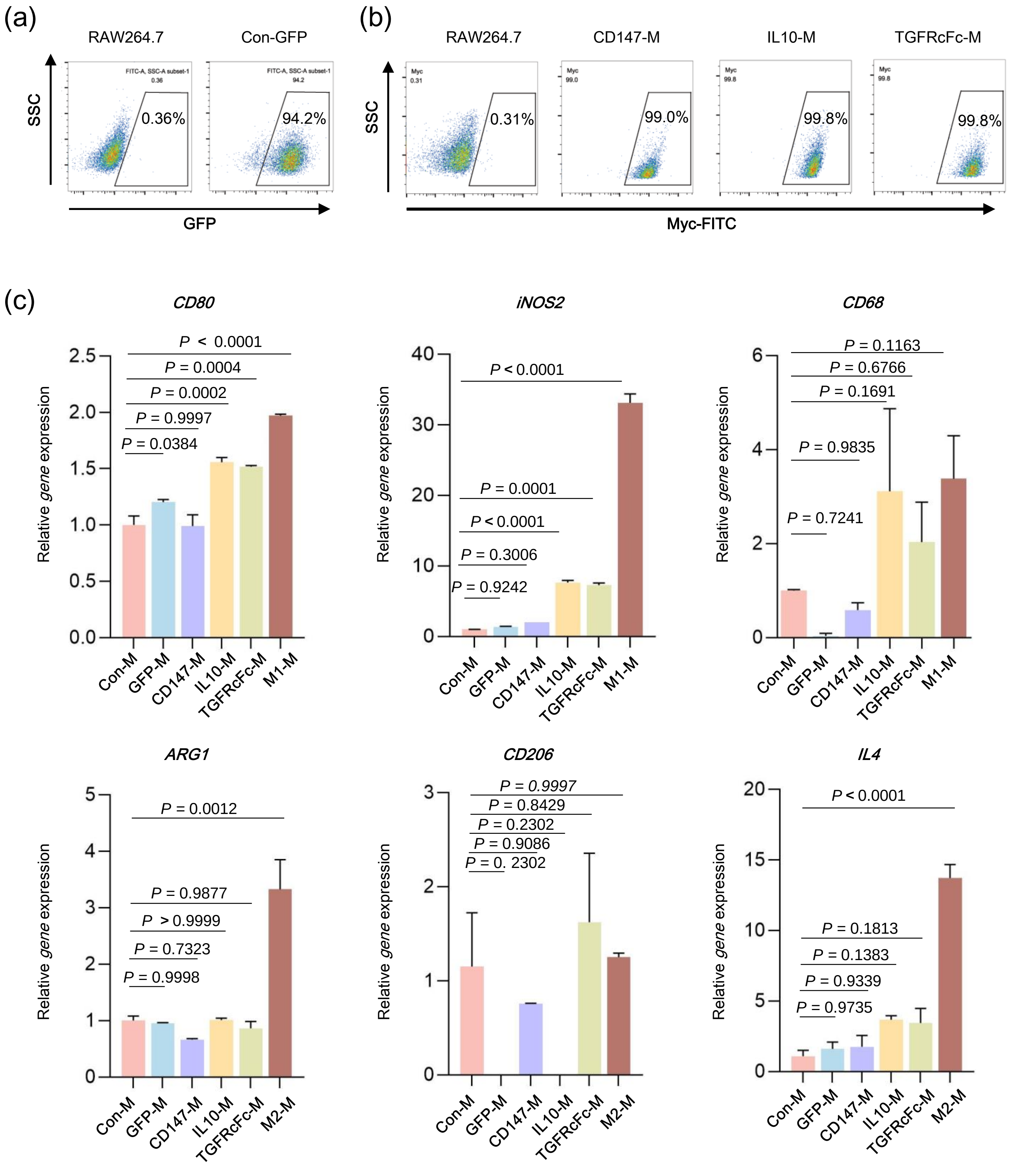

Figure S1 (a) Representative flow cytometry analysis of GFP+ cells. (b) Representative flow cytometry analysis of engineered cells by using a Myc-FITC antibody. (c) The mRNA levels of indicted gens in engineered cells were analyzed by RT-PCR (qPCR). All experiments were performed at least 3 times. Bars indicate the means ± standard error of the means.

Figure S2

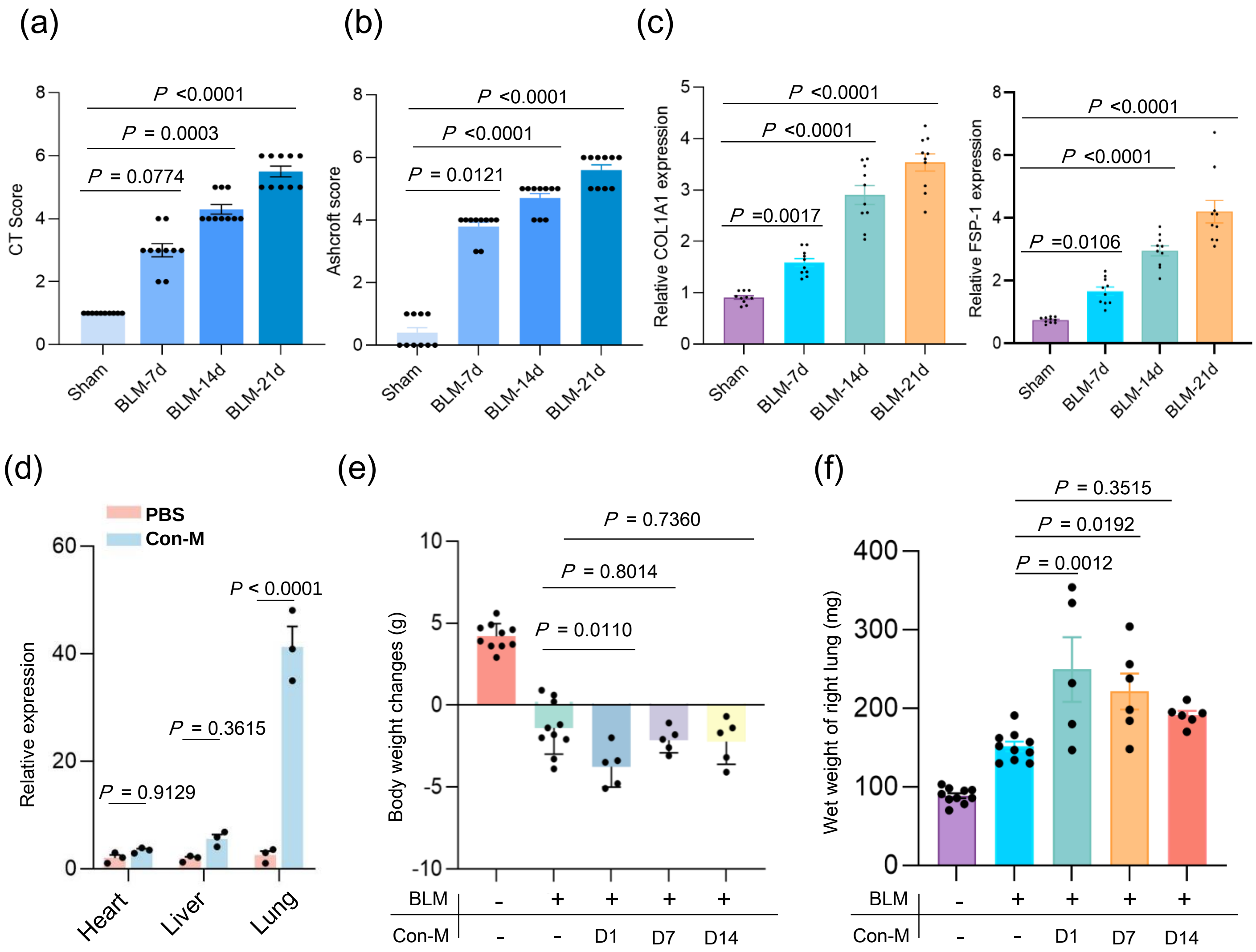

Figure S2 (a) CT fibrosis scores are presented on the day after BLM treated. (b) Ashcroft Score of lung sections are presented on the day after BLM treated. Body weight at day 21 after BLM treatment. (c) The mRNA levels of *COL1A1* and *FSP-1* in lungs on the day after BLM treated were analysed by RT-PCR (qPCR) (d) The gDNA levels of *BSD* in lungs, liver and heart from the mice infused with Con-M or not at the 5st day after BLM treated were analysed by RT-PCR (qPCR). (e) Body weight changes at day 21 after BLM treatment. (f) Right lung wet weight of mice from the indicated groups (n = 8-10). All experiments were performed at least three times. Bars indicate the means ± standard error of the means.

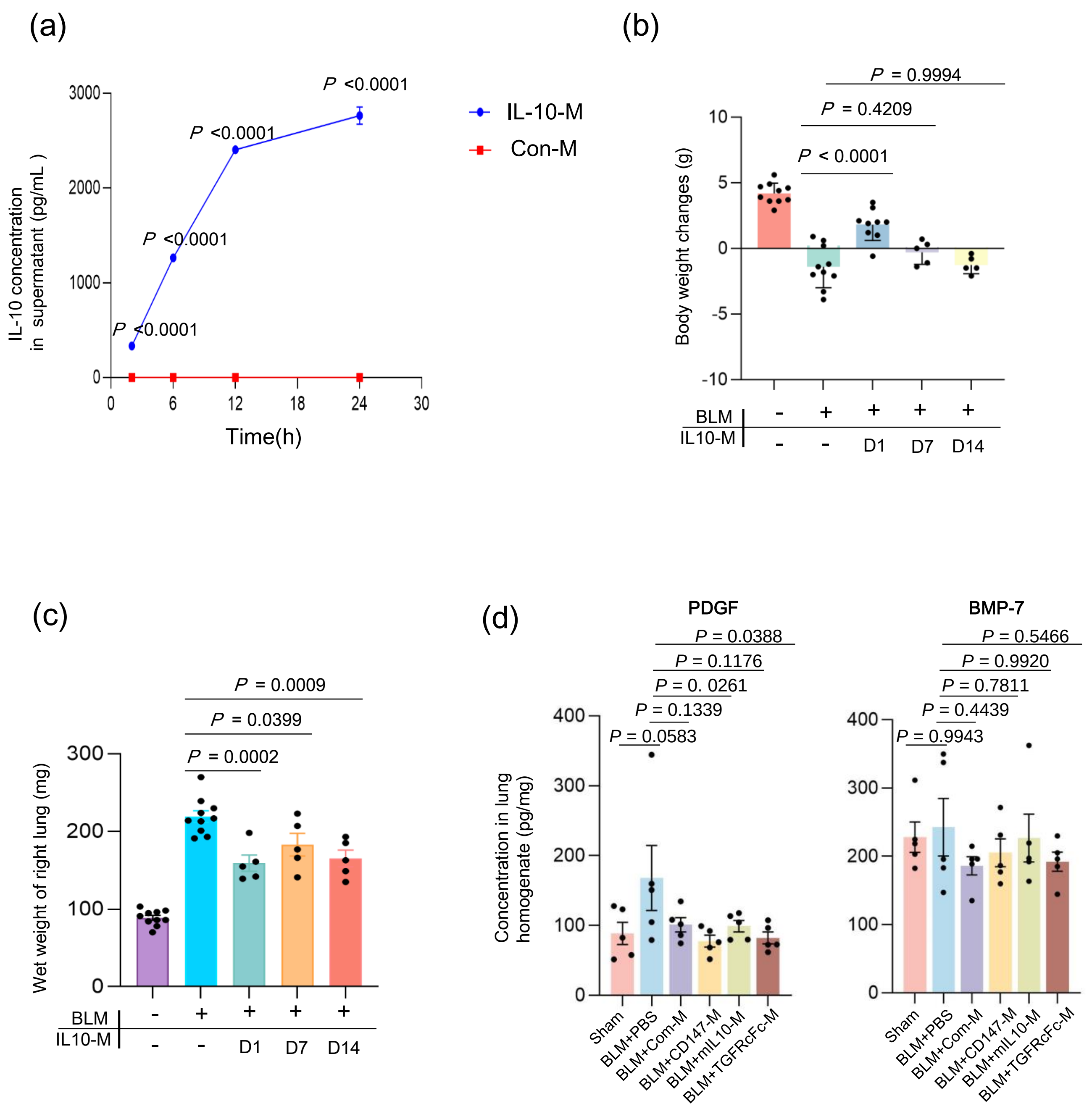

Figure S3 (a) The level of IL-10 in the supernatant of IL-10-M or Con-M was determined by ELISA. (b) Changes in body weight between day 21 and day 0. (c) Right lung wet weight of mice from the indicated groups (n = 8-10). (d) The concentrations of indicated cytokines were determined in lung homogenates after 5 days of BLM treatment by ELISA. All experiments were performed at least three times. Bars indicate the means ± standard error of the means.

Figure S4

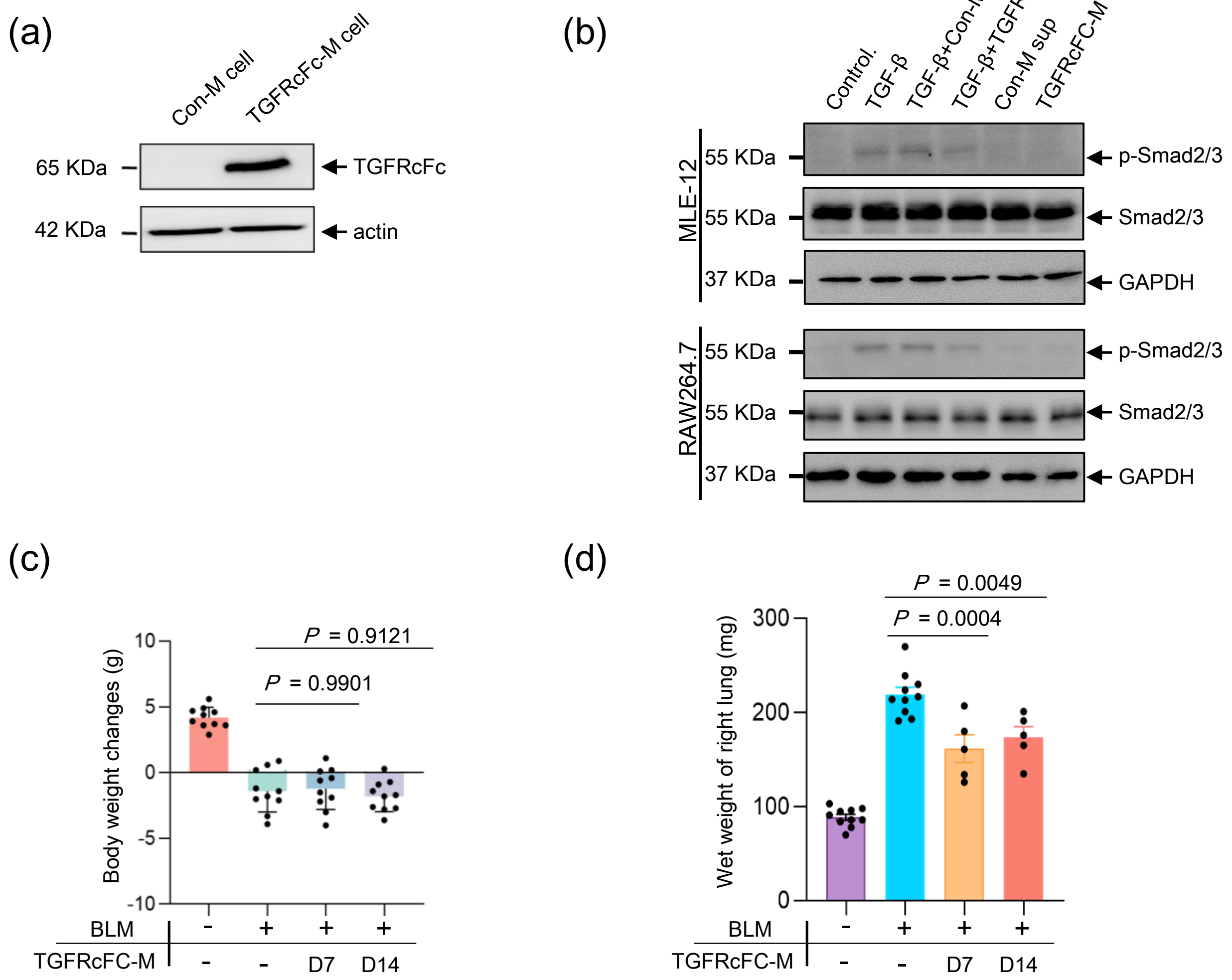

Figure S4 (a) Western blot analysis of TGFRcFC protein in TGFRcFC and control RAW264.7 cells. (b) TGFRcFC-M supernatant inhibit the TGF-β/Smad signalling pathway. MLE and RAW264.7 cells were treated as indicated and the cells collected and Western blot analysis was used to detected p-Smad2/3 and Smad2/3. (c) Changes in body weight between day 21 and day 0. (d) Right lung wet weight of mice from the indicated groups (n = 8-10). All experiments were performed at least 3 times. Bars indicate the means ± standard error of the means.

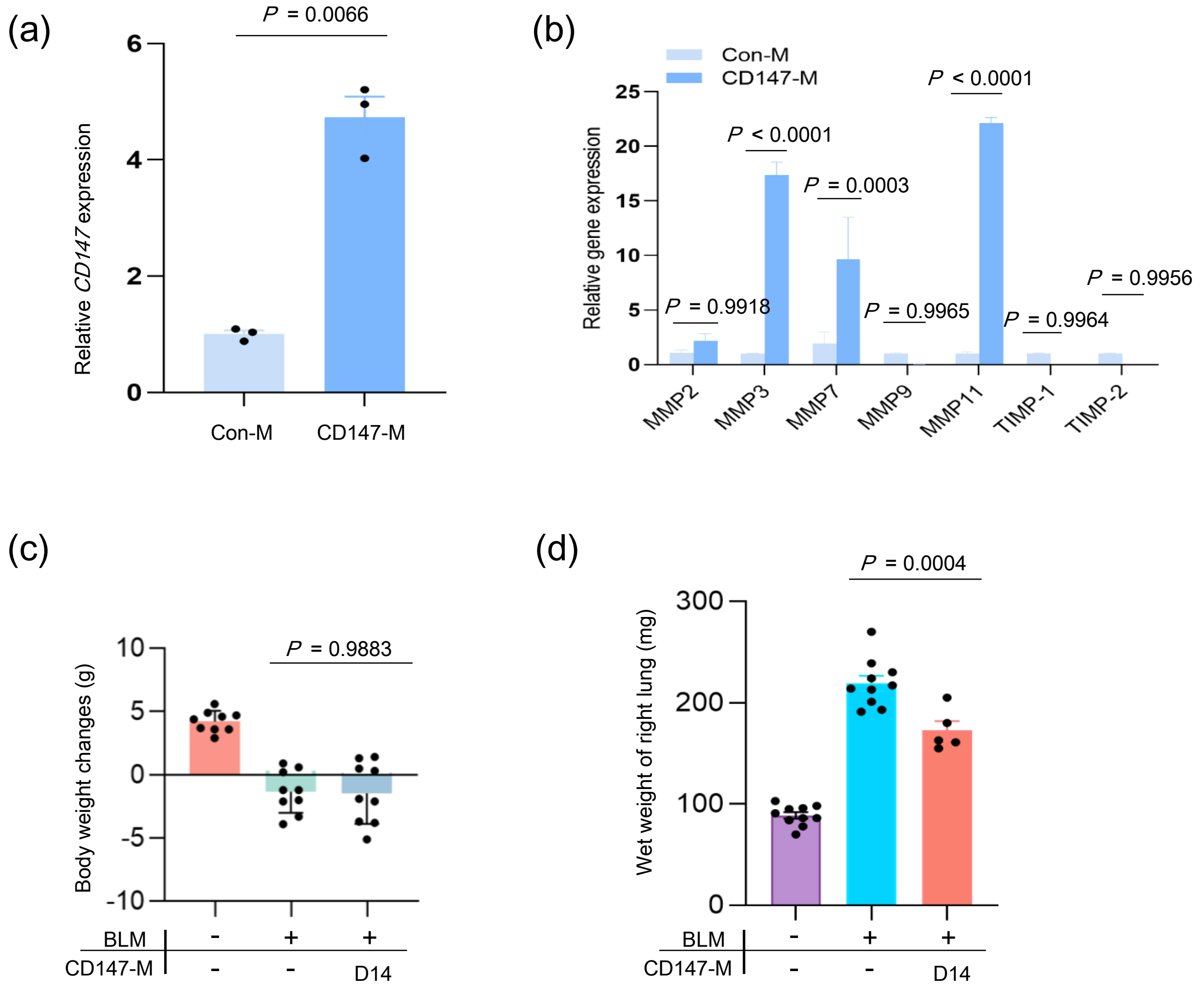

Figure S5 (a) mRNA levels of CD147 or (b) MMPs in CD147-M or Con-M cells were analysed by RT-PCR. (c) Changes in body weight between day 21 and day 0. (d) Right lung wet weight of mice from indicated groups (n = 8-10). All experiments were performed at least three times. Bars indicate the means ± standard error of the means.

Figure S6

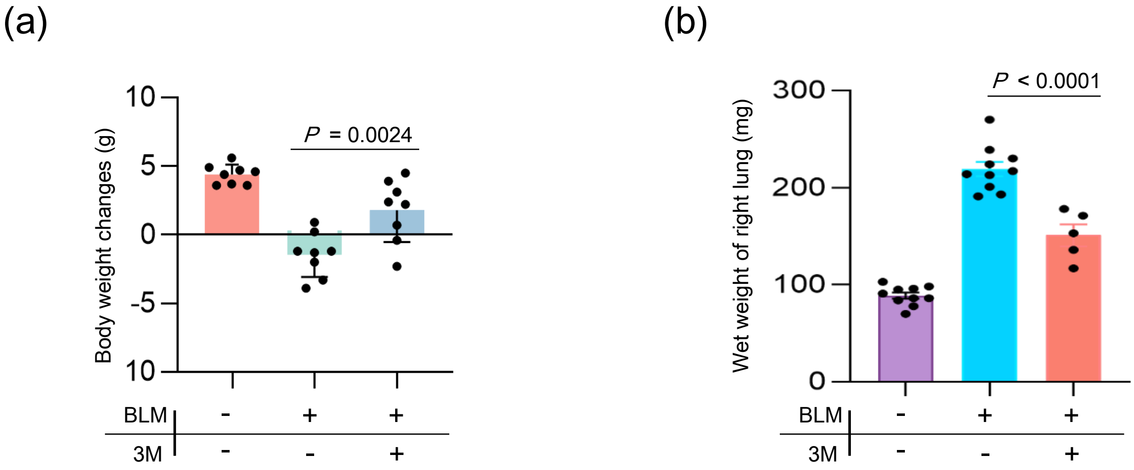

Figure S6 (a) Changes in body weight between day 21 and day 0. (b) Wet weight of right lung from mice in the indicated groups (n = 8-10). Results are representative of at least three independent experiments. Bars indicate the means ± standard error of the means.

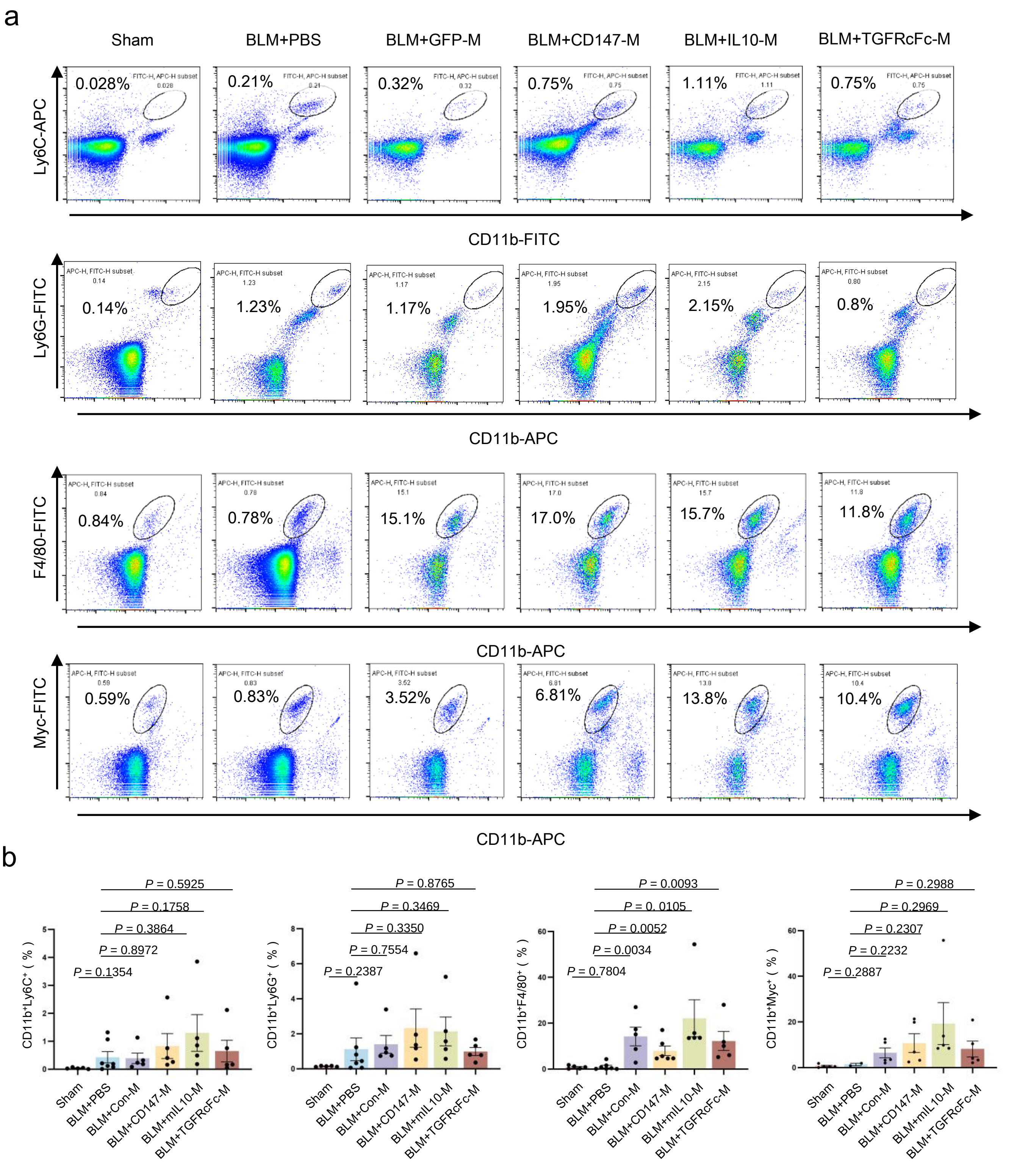

Figure S7 (a) Representative flow cytometry analysis of BAL cells from mice after 3 days the indicated cells were infused. (b) Total flow cytometry analysis of BAL cells from mice as (a). Bars indicate the means ± standard error of the means. Results are representative of at least three independent experiments.
